# Supplementary material for: US primary care in 2029: A Delphi survey on the impact of machine learning
Source: PLoS One. 2020 Oct 8;15(10):e0239947. doi: 10.1371/journal.pone.0239947 (PMC7544100; doi:10.1371/journal.pone.0239947)
Supplement: S1 Appendix — (PDF) [file pone.0239947.s001.pdf]

Primary Care in 2029: A Delphi Survey on the Impact of Machine Learning

---

Welcome

INFORMATION

Thank you for your interest in participating in this online survey. We are a team of researchers based at Open Notes, Beth Israel Deaconess Medical Center; Computational Health Informatics Program, Boston Children’s Hospital, and the School of Psychology, University of Plymouth. We are investigating the impact of machine learning and artificial intelligence on the future of primary care. You are invited to participate in this Delphi Poll as one of a panel of selected leading experts in machine learning, health informatics, medicine, and/or related fields.

The aim of this Delphi survey is forecast the impact of AI/machine learning technologies on primary care in 2029. Specifically, we are requesting your forecasts about how you believe these technologies will affect (if at all) the quality of patient care; access to care; and the composition of the primary care workforce by 2029. Your answers will help to inform debates about the inclusion of information about machine learning in medical curricula, and policy issues pertaining to the delivery of primary care.

OUR TEAM

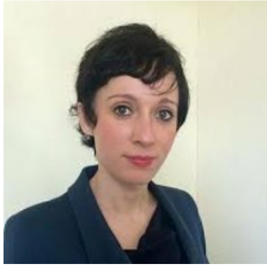

Dr. Charlotte Blease

Research Fellow

Beth Israel Deaconess Medical Center, Harvard Medical School

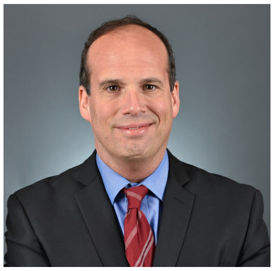

Prof. Ken Mandl

Director of CHIP

Computational Health Informatics Program
Boston Children's Hospital, Harvard Medical School

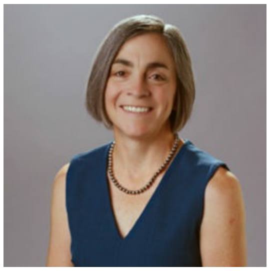

Dr. Catherine DesRoches

Executive Director of OpenNotes

Beth Israel Deaconess Medical Center, Harvard Medical School

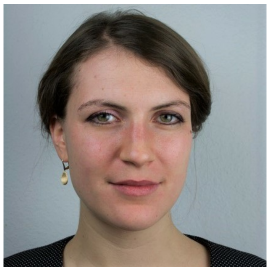

Dr. Cosima Locher

Research Fellow

Boston Children's Hospital, Harvard Medical School
University of Plymouth, UK

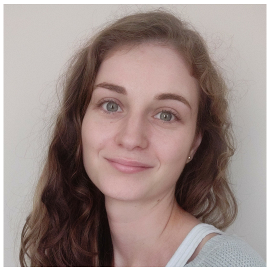

Anna Kharko

PhD Student

School of Psychology
University of Plymouth, UK

Should you have any questions about the survey or about our research, please contact Charlotte Blease using the email below:

DelphiAI2029@gmail.com

About the Survey

This Delphi Poll will involve **three rounds** of surveys, taking no more than **15 minutes each**.

This first round will consist of several open-ended questions asking you to predict how – if at all – AI/machine learning will impact primary care ten years from now. We are interested in your expert opinions about how AI/machine learning may impact diagnostic accuracy; health care disparities; and access to care, by 2029. We will also ask you to forecast how, ten years from now, AI/machine learning will affect the composition of the primary care workforce. In the first round we will also ask you some demographic questions, including about your professional background. **Round One should take around 10 minutes to complete.**

The answers provided by Delphi panelists in the first round will be transformed into statements. These statements will be sent out anonymously in the second round, to assess consensus amongst expert opinion. In the third and final round, statements that did not reach consensus will again be sent out anonymously and you will be invited to amend your answer (should you wish to do so) against the average response of Delphi panelists.

Time and Commitment of Participants

We will ensure that there is adequate time for panelists to provide their responses between rounds. Although this survey is not very demanding of time, the quality of Delphi Polls is dependent on a high level of participation between rounds. Therefore, should you be willing to participate, we request that you consider whether you can commit to responding to each of the three survey rounds.

All the data is confidential. This study has been given ethical approval by Beth Israel Deaconess Medical Center, Boston, and the University of Plymouth, UK. The reference number for this study is 2019P000564.

Do I have to take part?

Participation is voluntary. You may withdraw at any point during the questionnaire for any reason, before submitting your answers, by closing the browser.

How will your data be used?

The data we gather will be stored in a password-protected file and will be used to inform future academic publications. The data will be stored for a minimum of ten years after publication or public release. Delphi panelists will also be asked whether they wish to remain anonymous or agree to their name being published in a journal article upon which the aggregate data will be

based. It will not be possible to link data to individual participants.

Who will have access to your data?

Online Surveys (https://www.onlinesurveys.ac.uk/) is the data controller. You can read about their security policies here: https://www.onlinesurveys.ac.uk/help-support/online-surveys-security/. The information will not be shared with anyone other than members of our research team and will only be used to inform our project. Only members of the study team will be given access to the confidential data for monitoring and/or audit of the study to ensure we are complying with guidelines, or as otherwise required by law.

What if there is a problem?

If you have a concern about any aspect of this project, please speak to Dr Catherine DesRoches (cdesroch@bidmc.harvard.edu) or Dr Charlotte Blease (cblease@bidmc.harvard.edu) who will do their best to answer your query. The researcher should acknowledge your concern within 10 working days and give you an indication of how they intend to deal with it.

Under these conditions, do you agree to take part? ✱ *Required*

- ○ Yes, I agree to take part.
- ○ No, I don't agree to take part.

About You

First Name & Last Name ✱ *Required*

Individual survey responses are **confidential and anonymous**. Upon aggregating the data in a journal article, we may list the names of our Delphi experts. Do you permit your name being used as one of the respondents in a potential publication? ✱ *Required*

- Yes
- No

Email: ✱ *Required*

Please enter a valid email address.

Gender ✱ *Required*

- Female
- Male
- Transgender
- Other
- Prefer not to answer

If you selected Other, please specify:

Year of Birth ✱ *Required*

Nationality

Ethnicity

- American Indian or Alaskan Native
- Asian
- Black or African American
- Hispanic
- Native Hawaiian or other Pacific Islander
- White
- Mixed
- Other

If you selected Other, please specify:

Do you hold a MD? *Please select all that apply.* ✱ *Required*

- Yes
- No

Please enter medical specialty.

Do you hold a PhD? *Select all that apply.* ✱ *Required*

- Yes
- No

Please select subject area.

- Biology/Physiology/Neuroscience
- Computer Science/Informatics
- Engineering (non-Computer Science)
- Mathematics
- Medicine/Public Health
- Physics
- Philosophy
- Psychology/Cognitive Science
- Other

If you selected Other, please specify:

Current job. *Please select all that apply.* ✱ *Required*

- Industry
- Academia
- Medicine

☐ Other

If you selected Other, please specify:

What will happen?

The questions below request you to predict what you believe **will happen** and ***not what you personally*** would like to see happen.

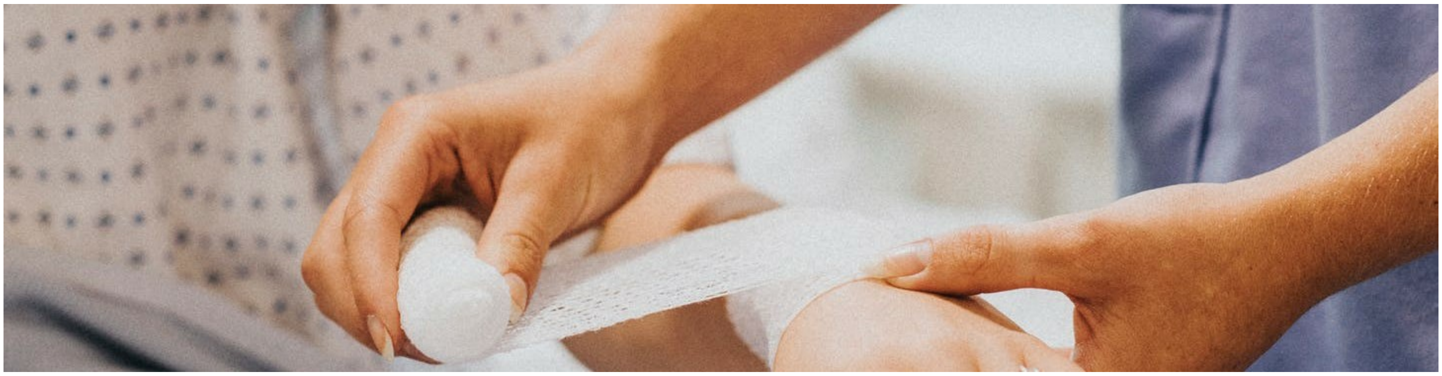

PART I: PATIENT CARE

By 2029, in your opinion, please predict the effect(s) – if any – of machine learning/AI on diagnostic accuracy in primary care in the USA. *Please describe 1 or 2 predictions, briefly, below.*

By 2029, in your opinion, please predict the effect(s) – if any – of machine learning/AI on health care disparities in the USA. *Please describe 1 or 2 predictions, briefly, below.*

By 2029, in your opinion, please predict the effects – if any – of machine learning/AI on the empathic care of primary care patients in the USA. *Please describe 1 or 2 predictions, briefly, below.*

What will happen?

The questions below request you to predict what you believe **will happen** and ***not what you personally*** would like to see happen.

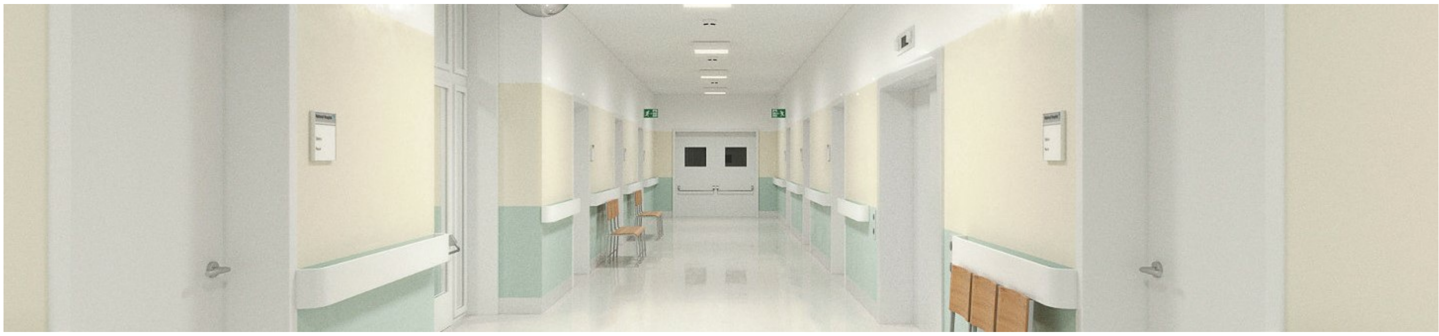

Part II: Access to care

By 2029, in your opinion, please predict the effects - if any - of machine learning/AI on patient access to medical care in the USA. *Please describe 1 or 2 predictions, briefly, below.*

What will happen?

The questions below request you to predict what you believe **will happen** and ***not what you personally*** would like to see happen.

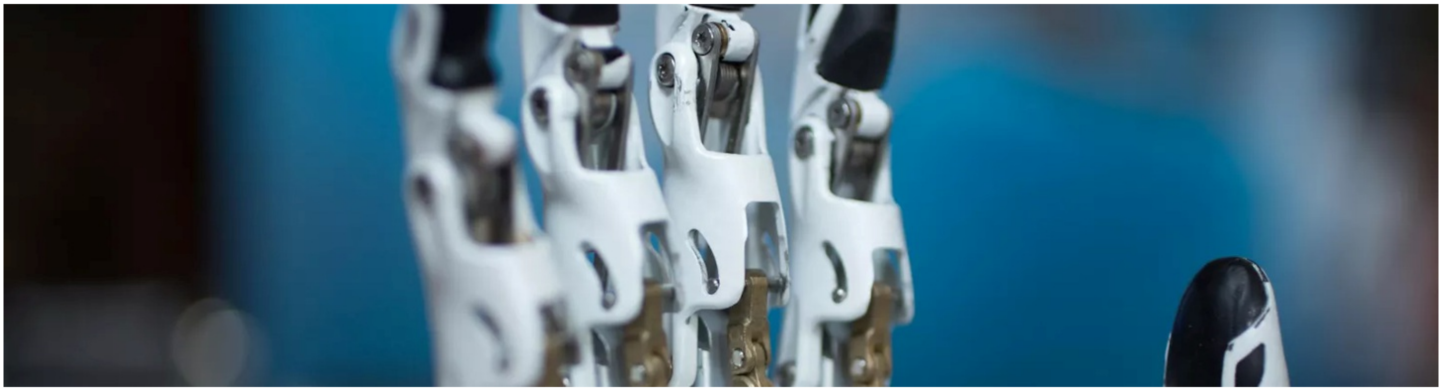

Part III: Primary Care Workface

By 2029, in your opinion, please predict the effects – if any – of machine learning/AI on the composition of the primary care workforce in the USA. *Please describe 1 or 2 predictions, briefly, below.*

What will happen?

The questions below request you to predict what you believe **will happen** and ***not what you personally*** would like to see happen.

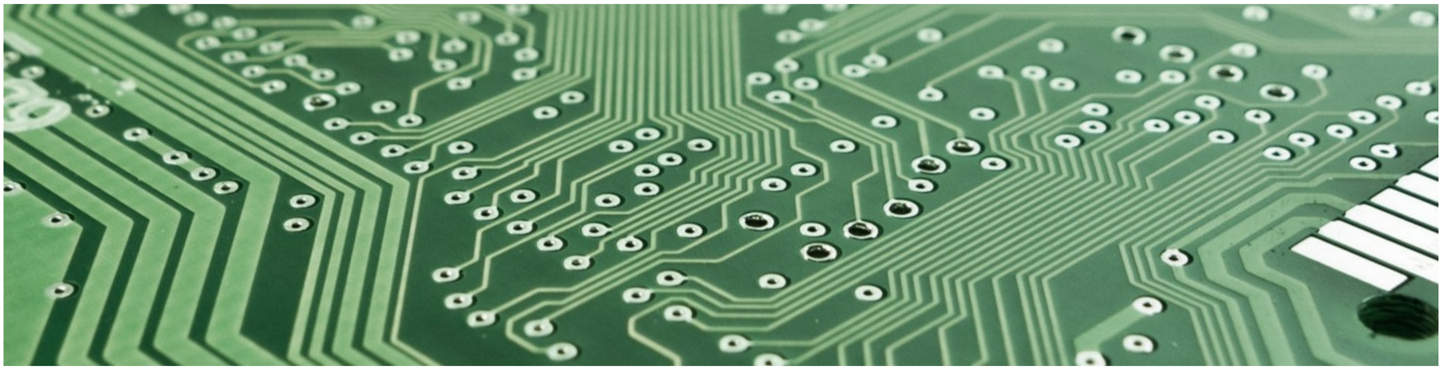

Part IV: Technological Advancements

In your opinion, please predict what - if any - major AI breakthroughs would be important to improve diagnostic accuracy in medicine? *Please provide **at least one or two** important breakthroughs, or if you believe no such breakthroughs are necessary, please elaborate.*

What will happen?

The questions below request you to predict what you believe **will happen** and ***not what you personally*** would like to see happen.

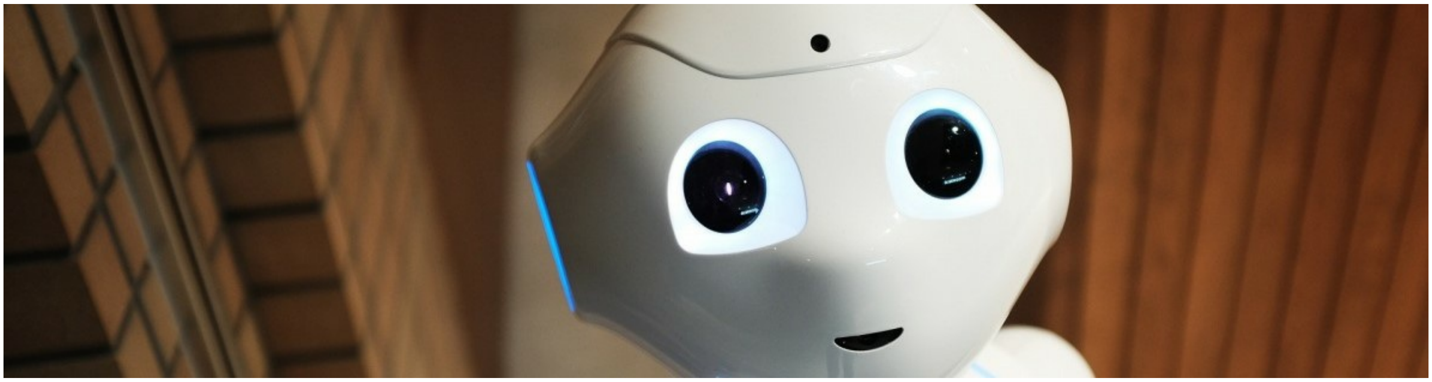

Part V: The Future of Primary Care Physicians

In your opinion, will primary care doctors ever become obsolete?

- Yes
- No
- Don't know

*If you answered ‘yes’, please give your best forecast of **how many years** from now this might happen. Please also provide a brief reason for your estimate.*

If you answered ‘no’, please elaborate.

If you answered ‘don't know’, please elaborate.

Questions & Comments

Feedback

Do you have any questions for the research team or comments about the topic of the survey? *If you do, please share them below. Otherwise, please **click 'Finish'.***

Final page

THANK YOU

We will collate the answers from all Delphi participants. This will inform the second round of the survey which will be sent in late October.

Thank you from the Study Team

(Ken Mandl, Catherine Desroches, Charlotte Blease, Cosima Locher, Anna Kharko).

If you have any questions, comments or concerns, please email DelphiAI2029@gmail.com

---

The photographic materials used in this survey are sourced from pexels.com

---
