# Supplementary material for: US primary care in 2029: A Delphi survey on the impact of machine learning
Source: PLoS One. 2020 Oct 8;15(10):e0239947. doi: 10.1371/journal.pone.0239947 (PMC7544100; doi:10.1371/journal.pone.0239947)
Supplement: S2 Appendix — (PDF) [file pone.0239947.s002.pdf]

Primary Care in 2029: A Delphi Survey on the Impact of Machine Learning

Welcome

INFORMATION

Thank you for your taking part in Round One of this Delphi Poll on the impact of AI/ML on primary care by 2019. We were very pleased with the response rate, and appreciate you taking the time to provide your insights. Your participation in Round Two will help us to further refine the collective predictions gathered in Round One.

Round Two of the survey involves **short close-ended questions**. These questions were generated from the combined responses in Round One. The survey has been pre-tested and should take **no more than 7 minutes** to complete.

In the third and final round, answers with less agreement among participants will again be sent out anonymously.

Thank you for lending us your expertise. It will not be possible to complete this study without you.

OUR TEAM

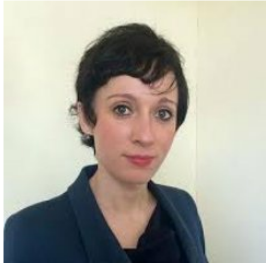

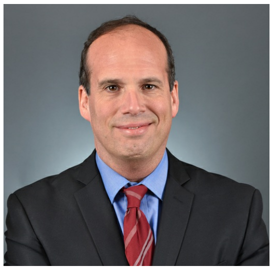

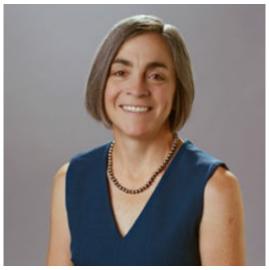

Dr. Charlotte Blease

Research Fellow

Beth Israel Deaconess Medical Center, Harvard Medical School

Prof. Ken Mandl

Director of CHIP

Computational Health Informatics Program
Boston Children's Hospital, Harvard Medical School

Dr. Catherine DesRoches

Executive Director of OpenNotes

Beth Israel Deaconess Medical Center, Harvard Medical School

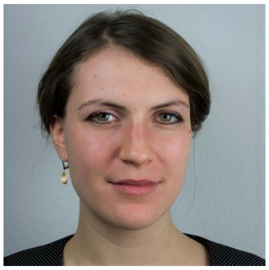

Dr. Cosima Locher

Research Fellow

Boston Children's Hospital, Harvard Medical School
University of Plymouth, UK

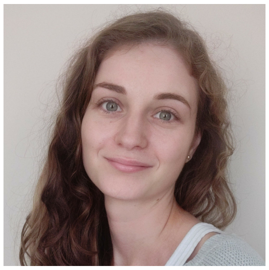

Anna Kharko

PhD Student

School of Psychology
University of Plymouth, UK

Should you have any questions about the survey or about our research, please contact Charlotte Blease using the email below:

DelphiAI2029@gmail.com

About the Survey

This survey asks you to predict how – if at all – AI/machine learning will impact primary care ten years from now. We are interested in your expert opinions about how AI/machine learning may impact diagnostic accuracy; health care disparities; and access to care, by 2029. We will also ask you to forecast how, ten years from now, AI/machine learning will affect the composition of the primary care workforce.

Your answers will be confidential among the survey team, and anonymous to other participants.

Time and Commitment of Participants

We will ensure that there is adequate time for panelists to provide their responses between rounds. Although this survey is not very demanding of time, the quality of Delphi Polls is dependent on a high level of participation between rounds. **This round will take around 7 minutes to complete. We anticipate that the third and final round will take no more than 5 minutes to complete.**

All the data is confidential. This study has been given ethical approval by Beth Israel Deaconess Medical Center, Boston, and the University of Plymouth, UK. The reference number for this study is 2019P000564.

Do I have to take part?

Participation is voluntary. You may withdraw at any point during the questionnaire for any reason, before submitting your answers, by closing the browser.

How will your data be used?

The data we gather will be stored in a password-protected file and will be used to inform future academic publications. The data will be stored for a minimum of ten years after publication or public release. Delphi panelists will also be asked whether they wish to remain anonymous or agree to their name being published in a journal article upon which the aggregate data will be based. It will not be possible to link data to individual participants.

Who will have access to your data?

Online Surveys (https://www.onlinesurveys.ac.uk/) is the data controller. You can read about

their security policies here: https://www.onlinesurveys.ac.uk/help-support/online-surveys-security/. The information will not be shared with anyone other than members of our research team and will only be used to inform our project. Only members of the study team will be given access to the confidential data for monitoring and/or audit of the study to ensure we are complying with guidelines, or as otherwise required by law.

What if there is a problem?

If you have a concern about any aspect of this project, please speak to Dr Catherine DesRoches (cdesroch@bidmc.harvard.edu) or Dr Charlotte Blease (cblease@bidmc.harvard.edu) who will do their best to answer your query. The researcher should acknowledge your concern within 10 working days and give you an indication of how they intend to deal with it.

Under these conditions, do you agree to take part? ✱ *Required*

- ○ Yes, I agree to take part.
- ○ No, I don't agree to take part.

First Name & Last Name

PART I: DIAGNOSTIC ACCURACY

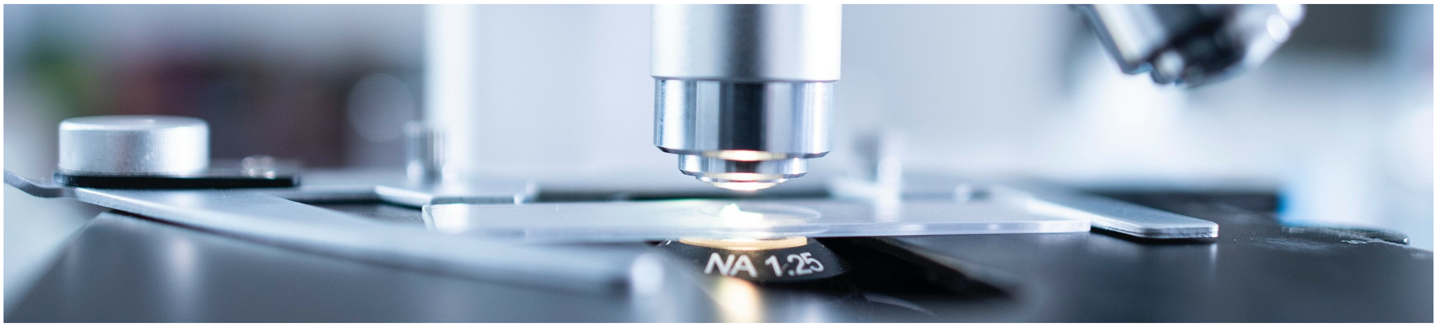

The questions below request you to predict what you believe **will happen** and ***not what you personally*** would like to see happen.

By 2029 in the US, as a result of AI/ML tools, rates of diagnostic accuracy ...

	1 - greatly decrease	2 - moderately decrease	3 - slightly decrease	4 - remain the same	5 - slightly increase	6 - moderately increase	7 - greatly increase
... will ...	☐	☐	☐	☐	☐	☐	☐
... for minority patients will ...	☐	☐	☐	☐	☐	☐	☐
... for some conditions where the markers of illness are relatively homogenous will ...	☐	☐	☐	☐	☐	☐	☐
... for rare conditions will …	☐	☐	☐	☐	☐	☐	☐

By 2029 in the US, as a result of AI/ML tools ...

	1 - greatly decrease	2 - moderately decrease	3 - slightly decrease	4 - remain the same	5 - slightly increase	6 - moderately increase	7 - greatly increase
… rates of overdiagnosis will…	☐	☐	☐	☐	☐	☐	☐
… rates of unnecessary testing will…	☐	☐	☐	☐	☐	☐	☐

By 2029 in the US, AI/ML-enabled tools will be routinely used ...

	1 - very unlikely	2 - moderately unlikely	3 - slightly unlikely	4 - uncertain	5 - slightly likely	6 - moderately likely	7 - very likely
... to assist doctors in diagnostic decision-making.	☐	☐	☐	☐	☐	☐	☐
... to assist primary care doctors with diagnosing the most difficult cases.	☐	☐	☐	☐	☐	☐	☐

... by patients to self-diagnose.	☐	☐	☐	☐	☐	☐	☐

By 2029 in the US,

	1- very unlikely	2 - moderately unlikely	3 - slightly unlikely	4 - uncertain	5 - slightly likely	6 - moderately likely	7 - very likely
... there will be a revamped nosology of many symptom-based disease categories.	☐	☐	☐	☐	☐	☐	☐

Do you have any comments on this part of the survey?

PART II: HEALTHCARE DISPARITIES

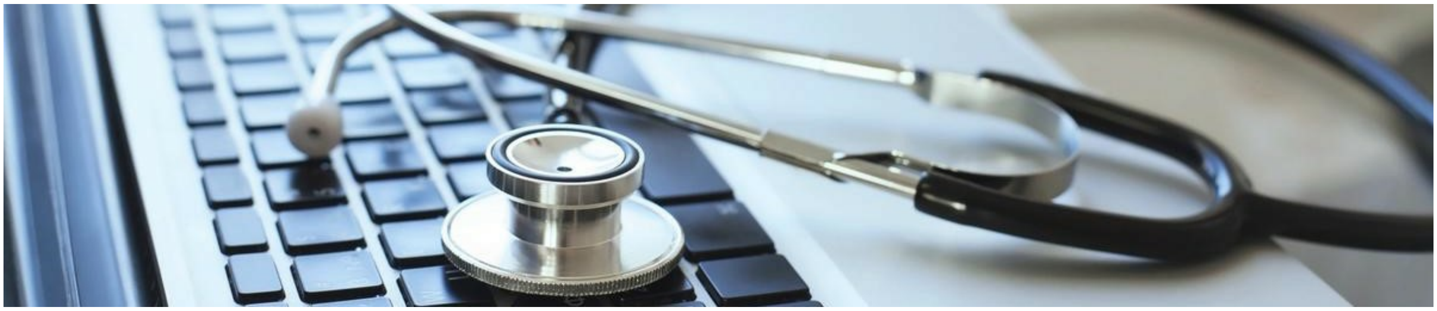

The questions below request you to predict what you believe **will happen** and ***not what you personally*** would like to see happen.

By 2029 in the US ...

	1 - greatly decrease	2 - moderately decrease	3 - slightly decrease	4 - remain the same	5 - slightly increase	6 - moderately increase	7 - greatly increase
... as a result of AI/ML enabled tools, healthcare disparities will ...	☐	☐	☐	☐	☐	☐	☐

By 2029 in the US ...

	1 - very unlikely	2 - moderately unlikely	3 - slightly unlikely	4 - uncertain	5 - slightly likely	6 - moderately likely	7 - very likely

… more sophisticated AI/ML resources will only be available to higher income individuals.	☐	☐	☐	☐	☐	☐	☐
… AI/ML tools will improve diagnostic accuracy for those with limited access to human experts.	☐	☐	☐	☐	☐	☐	☐
… private hospitals will have an advantage in using AI/ML resources to improve diagnostic accuracy compared to public hospitals.	☐	☐	☐	☐	☐	☐	☐
… there will be representative data collection among minority groups.	☐	☐	☐	☐	☐	☐	☐

Do you have any comments on this part of the survey?

PART III: EMPATHIC CARE OF PATIENTS

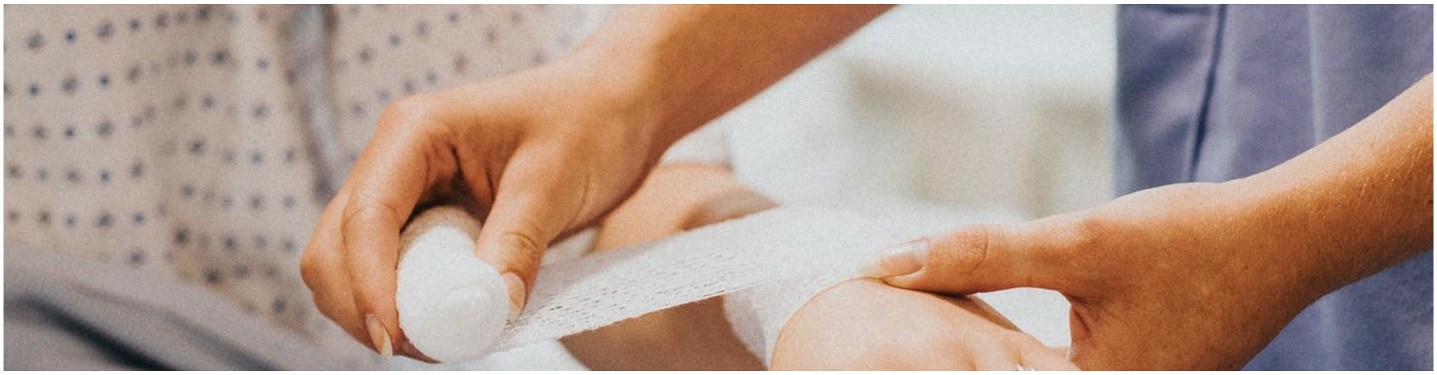

The questions below request you to predict what you believe **will happen** and ***not what you personally*** would like to see happen.

In primary care, by 2029 in the US, the availability of AI/ML tools mean that ...

	1 - greatly decrease	2 - moderately decrease	3 - slightly decrease	4 - remain the same	5 - slightly increase	6 - moderately increase	7 - greatly increase
… levels of empathic care will …	☐	☐	☐	☐	☐	☐	☐
... the total time patients spend with doctors will …	☐	☐	☐	☐	☐	☐	☐
… the documentation burden on doctors will …	☐	☐	☐	☐	☐	☐	☐

By 2029 in the US ...

	1 - very unlikely	2 - moderately unlikely	3 - slightly unlikely	4 - uncertain	5 - slightly likely	6 - moderately likely	7 - very likely
... AI/ML will offer direct resources for delivering empathic care.	☐	☐	☐	☐	☐	☐	☐
... health care will be increasingly productized.	☐	☐	☐	☐	☐	☐	☐
... AI/ML tools will help assist doctors in shared decision-making with patients.	☐	☐	☐	☐	☐	☐	☐
... AI/ML tools will help clinicians to think more about patients' lifestyle.	☐	☐	☐	☐	☐	☐	☐
... AI/ML tools will use data on the social determinants of health to devise personalized health plans.	☐	☐	☐	☐	☐	☐	☐

Do you have any comments on this part of the survey?

PART IV: ACCESS TO CARE

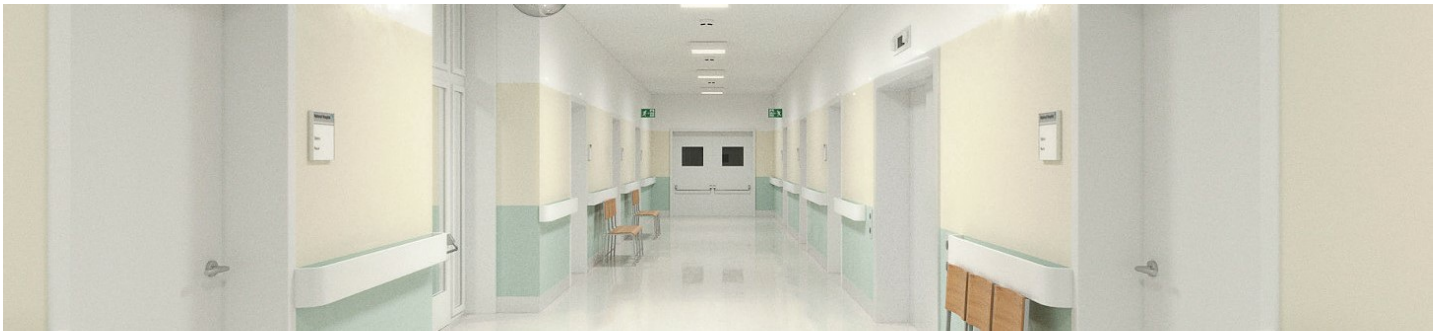

The questions below request you to predict what you believe **will happen** and ***not what you personally*** would like to see happen.

By 2029 in the US, as a result of AI/ML tools, patient access to ...

	1 - greatly decrease	2 - moderately decrease	3 - slightly decrease	4 - remain the same	5 - slightly increase	6 - moderately increase	7 - greatly increase
… medical care will ...	☐	☐	☐	☐	☐	☐	☐
... expert doctor knowledge will …	☐	☐	☐	☐	☐	☐	☐
… basic medical expertise via electronic devices will …	☐	☐	☐	☐	☐	☐	☐

By 2029 in the US ...

	1 - greatly decrease	2 - moderately decrease	3 - slightly decrease	4 - remain the same	5 - slightly increase	6 - moderately increase	7 - greatly increase
… the use of AI/ML patient triage tools by health organization will…	☐	☐	☐	☐	☐	☐	☐
... the use of telemedicine will…	☐	☐	☐	☐	☐	☐	☐

When it comes to the impact of AI/ML on patient access to medical care ...

	1 - very unlikely	2 - moderately unlikely	3 - slightly unlikely	4 - uncertain	5 - slightly likely	6 - moderately likely	7 - very likely
... the US will lag behind other developed countries.	☐	☐	☐	☐	☐	☐	☐

By 2029 in the US ...

	1 - very unlikely	2 - moderately unlikely	3 - slightly unlikely	4 - uncertain	5 - slightly likely	6 - moderately likely	7 - very likely

... AI/ML enabled resources will be too expensive for most patients.	☐	☐	☐	☐	☐	☐	☐
... AI/ML will be used for patient-doctor matching.	☐	☐	☐	☐	☐	☐	☐
... AI/ML will be used for appointment scheduling.	☐	☐	☐	☐	☐	☐	☐

Do you have any comments on this part of the survey?

PART V: PRIMARY CARE WORKFORCE

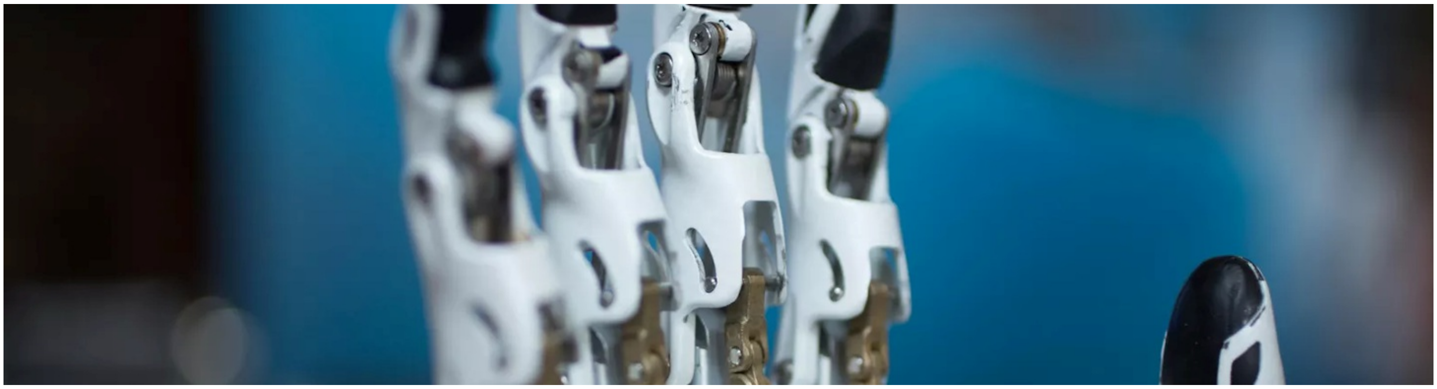

The questions below request you to predict what you believe **will happen** and ***not what you personally*** would like to see happen.

By 2029 in the US ...

	1 - greatly decrease	2 - moderately decrease	3 - slightly decrease	4 - remain the same	5 - slightly increase	6 - moderately increase	7 - greatly increase
… the proportion of mid-level clinicians (e.g. nurse practitioners) will …	☐	☐	☐	☐	☐	☐	☐

... the number of clinicians with degrees in engineering or computer science entering medicine will …	☐	☐	☐	☐	☐	☐	☐
… efficiency in the delivery of primary care will …	☐	☐	☐	☐	☐	☐	☐
… training requirements in working with AI/ML will …	☐	☐	☐	☐	☐	☐	☐

By 2029 in the US ...

	1 - very unlikely	2 - moderately unlikely	3 - slightly unlikely	4 - uncertain	5 - slightly likely	6 - moderately likely	7 - very likely
… AI/ML tools will enable clinicians with lower licenses to do higher-level jobs.	☐	☐	☐	☐	☐	☐	☐

… doctors will transition from the role of dispensers of knowledge to managing teams and information systems.	☐	☐	☐	☐	☐	☐	☐
… AI/ML tools will change the reimbursement structure for routine clinical tasks.	☐	☐	☐	☐	☐	☐	☐

Do you have any comments on this part of the survey?

PART VI: TECHNOLOGICAL ADVANCEMENTS IN PRIMARY CARE

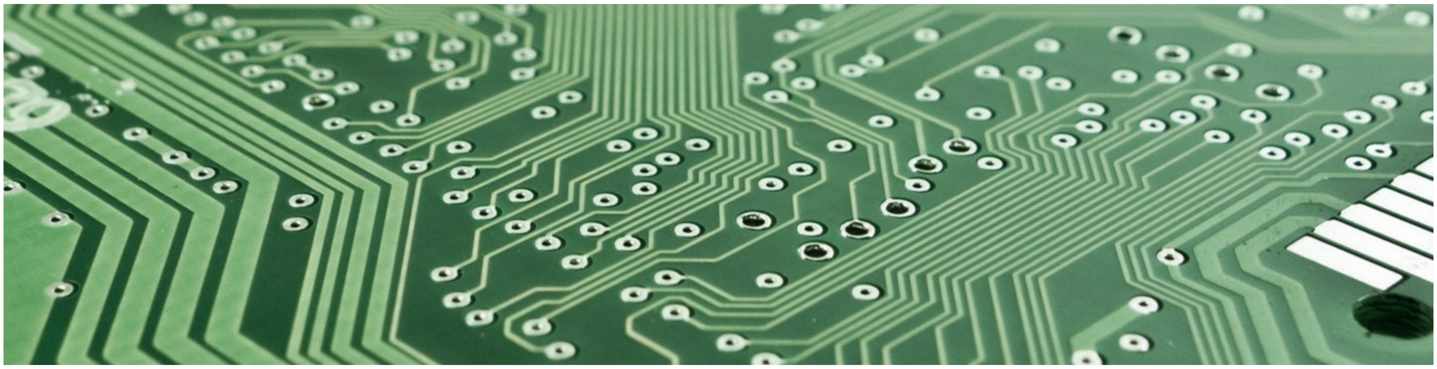

The questions below request you to predict what you believe **will happen** and ***not what you personally*** would like to see happen.

Will improvements in the diagnostic accuracy of AI/ML tools require technological breakthroughs?

- Yes
- No

Improvements in diagnostic accuracy of AI/ML tools will require technological breakthroughs ...

	1 - strongly disagree	2 - moderately disagree	3 - slightly disagree	4 - neutral	5 - slightly agree	6 - moderately agree	7 - strongly agree
… in causal modelling.	☐	☐	☐	☐	☐	☐	☐
… in artificial general intelligence.	☐	☐	☐	☐	☐	☐	☐

… in the interpretability of certain approaches such as deep learning.	☐	☐	☐	☐	☐	☐	☐
… in human-level natural language processing.	☐	☐	☐	☐	☐	☐	☐
… in semi-supervised learning.	☐	☐	☐	☐	☐	☐	☐
… to harness the sensor data from smartphones and wearables to forecast individual symptom trajectories.	☐	☐	☐	☐	☐	☐	☐

To improve the diagnostic accuracy of AI/ML tools, will require ...

	1 - strongly disagree	2 - moderately disagree	3 - slightly disagree	4 - neutral	5 - slightly agree	6 - moderately agree	7 - strongly agree
… improved data quality.	☐	☐	☐	☐	☐	☐	☐
… integrated data sets.	☐	☐	☐	☐	☐	☐	☐

In the US, regulatory issues in improving diagnostic accuracy of AI/ML tools ...

	1 - strongly disagree	2 - moderately disagree	3 - slightly disagree	4 - neutral	5 - slightly agree	6 - moderately agree	7 - strongly agree
... will be more challenging than technical issues.	☐	☐	☐	☐	☐	☐	☐

Do you have any comments on this part of the survey?

PART VII: THE LONG-TERM FUTURE OF THE PROFESSION

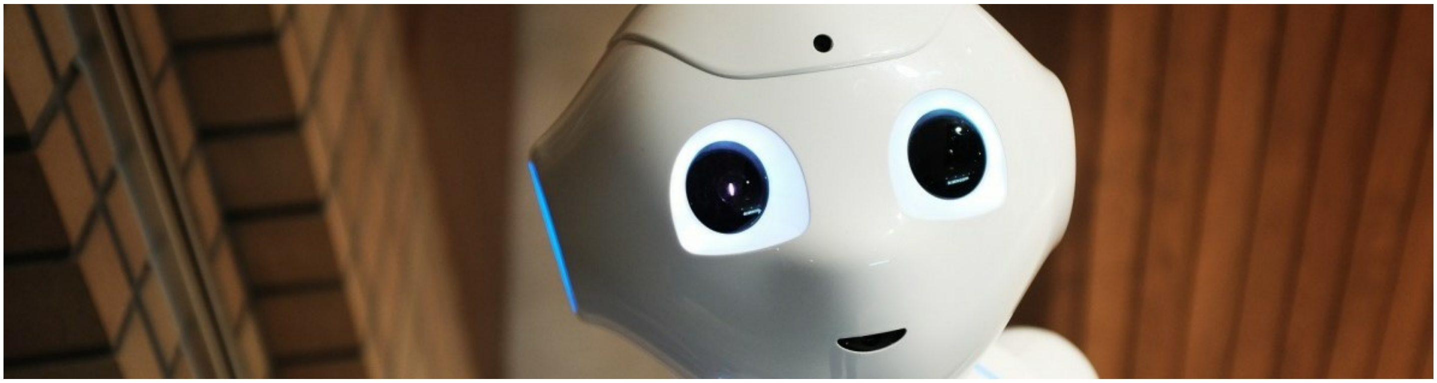

The questions below request you to predict what you believe **will happen** and ***not what you personally*** would like to see happen.

In the US ...

	1 - strongly disagree	2 - moderately disagree	3 - slightly disagree	4 - neutral	5 - slightly agree	6 - moderately agree	7 - strongly agree
... primary care doctors will be one of the last specialties to be replaced by AI/ML in medicine.	☐	☐	☐	☐	☐	☐	☐
... primary care doctors will always be required as gatekeepers in medicine.	☐	☐	☐	☐	☐	☐	☐

... primary care doctors will always be required to synthesize information.	☐	☐	☐	☐	☐	☐	☐
... primary care doctors will always be required to deliver empathic aspects of care.	☐	☐	☐	☐	☐	☐	☐
... patients will always prefer humans as gatekeepers of their medical care.	☐	☐	☐	☐	☐	☐	☐
... adoption of AI/ML tools in health care will be slow due to the culture of medicine.	☐	☐	☐	☐	☐	☐	☐

In the US, there is a 90% chance that primary care doctors will be obsolete ...

	1 - strongly disagree	2 - moderately disagree	3 - slightly disagree	4 - neutral	5 - slightly agree	6 - moderately agree	7 - strongly agree
… 50 years from now.	☐	☐	☐	☐	☐	☐	☐

… 100 years from now.	☐	☐	☐	☐	☐	☐	☐

Do you have any comments on this part of the survey?

Questions & Comments

FEEDBACK

Do you have any questions for the research team or comments about the topic of the survey? *If you do, please share them below. Otherwise, please* ***click 'Finish'.***

Final page

THANK YOU

We will collate the answers from all Delphi participants. This will inform the second round of the survey which will be sent in late October.

Thank you from the Study Team

(Ken Mandl, Catherine Desroches, Charlotte Blease, Cosima Locher, Anna Kharko).

If you have any questions, comments or concerns, please email DelphiAI2029@gmail.com

---

The photographic materials used in this survey are sourced from pexels.com

---
