# Supplementary material for: US primary care in 2029: A Delphi survey on the impact of machine learning
Source: PLoS One. 2020 Oct 8;15(10):e0239947. doi: 10.1371/journal.pone.0239947 (PMC7544100; doi:10.1371/journal.pone.0239947)
Supplement: S3 Appendix — (PDF) [file pone.0239947.s003.pdf]

Appendix 3. Round 2 De-identified Raw Data

| **Section** | ***Question*** | **Scale** | **A** | **B** | **C** | **D** | **E** | **F** | **G** | **H** | **I** | **J** | **K** | **L** | **M** |
|---|---|---|---|---|---|---|---|---|---|---|---|---|---|---|---|
| I. Diagnostic Accuracy | *By 2029 in the US, as a result of AI/ML tools, rates of diagnostic accuracy will …* | 1 - greatly decrease to 7 - greatly increase | 5 | 6 | 6 | 5 | 6 | 6 | 5 | 5 | 5 | 5 | 7 | 7 | 4 |
| | *By 2029 in the US, as a result of AI/ML tools, rates of diagnostic accuracy for minority patients will …* | | 4 | 6 | 6 | 4 | 5 | 6 | 6 | 5 | 4 | 5 | 6 | 6 | 4 |
| | *By 2029 in the US, as a result of AI/ML tools, rates of diagnostic accuracy for some conditions where the markers of illness are relatively homogenous will …* | | 6 | 6 | 6 | 5 | 6 | 6 | 6 | 5 | 4 | 5 | 7 | 7 | 5 |
| | *By 2029 in the US, as a result of AI/ML tools, rates of diagnostic accuracy for rare conditions will …* | | 4 | 6 | 6 | 6 | 5 | 5 | 6 | 7 | 5 | 5 | 5 | 6 | 4 |
| | *By 2029 in the US, as a result of AI/ML tools, rates of overdiagnosis will …* | | 5 | 4 | 2 | 4 | 6 | 2 | 5 | 5 | 4 | 3 | 2 | 3 | 5 |
| | *By 2029 in the US, as a result of AI/ML tools, rates of unnecessary testing will …* | | 5 | 5 | 2 | 4 | 6 | 3 | 5 | 3 | 4 | 3 | 2 | 2 | 5 |
| | *By 2029 in the US, AI/ML-enabled tools will be routinely used to assist doctors in diagnostic decision-making.* | 1 - very unlikely to 7 - very likely | 5 | 6 | 6 | 5 | 6 | 7 | 6 | 5 | 4 | 5 | 7 | 7 | 2 |
| | *By 2029 in the US, AI/ML-enabled tools will be routinely used to assist primary care doctors with diagnosing the most difficult cases.* | | 5 | 7 | 7 | 5 | 5 | 6 | 6 | 7 | 5 | 3 | 6 | 7 | 3 |
| | *By 2029 in the US, AI/ML-enabled tools will be routinely used by patients to self-diagnose.* | | 6 | 5 | 5 | 5 | 5 | 7 | 6 | 7 | 2 | 6 | 5 | 6 | 5 |
| | *By 2029 in the US, there will be a revamped nosology of many symptom-based disease categories.* | | 5 | 6 | 6 | 5 | 7 | 7 | 4 | 5 | 5 | 5 | 5 | 5 | 4 |

| | | | | | | | | | | | | | | | |
|---|---|---|---|---|---|---|---|---|---|---|---|---|---|---|---|
| II. Healthcare Disparities | *By 2029 in the US, as a result of AI/ML enabled tools, healthcare disparities will ...* | 1 - greatly decrease to 7 - greatly increase | 4 | 4 | 4 | 5 | 5 | 6 | 4 | 4 | 4 | 3 | 3 | 4 | 4 |
| | *By 2029 in the US, more sophisticated AI/ML resources will only be available to higher income individuals.* | 1 - very unlikely to 7 - very likely | 3 | 7 | 2 | 5 | 6 | 7 | 5 | 1 | 6 | 6 | 5 | 4 | 5 |
| | *By 2029 in the US, AI/ML tools will improve diagnostic accuracy for those with limited access to human experts.* | | 4 | 6 | 3 | 4 | 6 | 6 | 5 | 5 | 4 | 5 | 6 | 6 | 4 |
| | *By 2029 in the US, private hospitals will have an advantage in using AI/ML resources to improve diagnostic accuracy compared to public hospitals.* | | 6 | 4 | 3 | 5 | 4 | 5 | 5 | 6 | 6 | 6 | 3 | 5 | 4 |
| | *By 2029 in the US, there will be representative data collection among minority groups.* | | 5 | 3 | 6 | 4 | 5 | 7 | 3 | 6 | 4 | 5 | 3 | 5 | 3 |
| III. Empathic Care of Patients | *In primary care, by 2029 in the US, the availability of AI/ML tools mean that levels of empathic care will …* | 1 - greatly decrease to 7 - greatly increase | 4 | 4 | 5 | 5 | 5 | 7 | 4 | 5 | 4 | 4 | 5 | 4 | 4 |
| | *In primary care, by 2029 in the US, the availability of AI/ML tools mean the total time patients spend with doctors will …* | | 3 | 4 | 4 | 5 | 4 | 4 | 3 | 4 | 4 | 5 | 5 | 3 | 4 |
| | *In primary care, by 2029 in the US, the availability of AI/ML tools mean the documentation burden on doctors will …* | | 5 | 3 | 3 | 3 | 4 | 4 | 5 | 3 | 4 | 3 | 3 | 3 | 4 |
| | *By 2029 in the US, AI/ML will offer direct resources for delivering empathic care.* | 1 - very unlikely to 7 - very likely | 6 | 2 | 5 | 5 | 5 | 4 | 1 | 4 | 2 | 1 | 3 | 2 | 5 |
| | *By 2029 in the US,  health care will be increasingly productized.* | | 6 | 6 | 3 | 4 | 6 | 1 | 5 | 5 | 5 | 6 | 3 | 7 | 6 |
| | *By 2029 in the US, AI/ML tools will help assist doctors in shared decision-making with patients.* | | 5 | 5 | 5 | 6 | 6 | 7 | 4 | 5 | 4 | 5 | 7 | 7 | 6 |
| | *By 2029 in the US, AI/ML tools will help clinicians to think more about patients' lifestyle.* | | 4 | 6 | 6 | 5 | 6 | 7 | 5 | 4 | 4 | 6 | 6 | 6 | 4 |
| | *By 2029 in the US, AI/ML tools will use data on the social determinants of health to devise personalized health plans.* | | 6 | 6 | 6 | 4 | 6 | 4 | 5 | 5 | 2 | 5 | 6 | 6 | 6 |

| | | | | | | | | | | | | | | | |
|---|---|---|---|---|---|---|---|---|---|---|---|---|---|---|---|
| IV. Access to Care | *By 2029 in the US, as a result of AI/ML tools, patient access to medical care will …* | 1 - greatly decrease to 7 - greatly increase | 5 | 5 | 5 | 5 | 6 | 7 | 4 | 5 | 4 | 5 | 5 | 6 | 4 |
| | *By 2029 in the US, as a result of AI/ML tools, patient access to expert doctor knowledge will …* | | 5 | 5 | 5 | 5 | 6 | 6 | 5 | 4 | 4 | 5 | 5 | 5 | 4 |
| | *By 2029 in the US, as a result of AI/ML tools, patient access to basic medical expertise via electronic devices will …* | | 5 | 6 | 6 | 5 | 7 | 6 | 5 | 6 | 4 | 5 | 5 | 5 | 7 |
| | *By 2029 in the US, the use of AI/ML patient triage tools by health organization will …* | | 5 | 6 | 6 | 5 | 6 | 6 | 6 | 5 | 6 | 5 | 5 | 7 | 5 |
| | *By 2029 in the US, the use of telemedicine will …* | | 6 | 7 | 6 | 5 | 6 | 7 | 7 | 6 | 7 | 6 | 6 | 6 | 6 |
| | *When it comes to the impact of AI/ML on patient access to medical care, the US will lag behind other developed countries.* | 1 - very unlikely to 7 - very likely | 5 | 5 | 2 | 4 | 3 | 7 | 2 | 1 | 4 | 5 | 3 | 5 | 4 |
| | *By 2029 in the US, AI/ML enabled resources will be too expensive for most patients.* | | 3 | 3 | 2 | 4 | 3 | 1 | 4 | 1 | 5 | 5 | 2 | 2 | 4 |
| | *By 2029 in the US, AI/ML will be used for patient-doctor matching.* | | 6 | 4 | 3 | 6 | 6 | 4 | 5 | 5 | 6 | 1 | 3 | 6 | 5 |
| | *By 2029 in the US, AI/ML will be used for appointment scheduling.* | | 7 | 5 | 5 | 6 | 7 | 7 | 6 | 5 | 7 | 6 | 2 | 5 | 7 |
| V. Primary Care Workforce | *By 2029 in the US, the proportion of mid-level clinicians (e.g. nurse practitioners) will …* | 1 - greatly decrease to 7 - greatly increase | 6 | 6 | 4 | 6 | 5 | 7 | 4 | 6 | 6 | 4 | 6 | 7 | 5 |
| | *By 2029 in the US, the number of clinicians with degrees in engineering or computer science entering medicine will …* | | 6 | 5 | 4 | 5 | 6 | 6 | 5 | 7 | 5 | 5 | 6 | 7 | 5 |
| | *By 2029 in the US, efficiency in the delivery of primary care will …* | | 5 | 4 | 5 | 4 | 5 | 6 | 5 | 6 | 5 | 5 | 6 | 6 | 4 |
| | *By 2029 in the US, training requirements in working with AI/ML will …* | | 5 | 5 | 6 | 3 | 5 | 5 | 7 | 6 | 5 | 5 | 6 | 6 | 4 |
| | *By 2029 in the US, AI/ML tools will enable clinicians with lower licenses to do higher-level jobs.* | 1 - very unlikely to 7 - very likely | 5 | 5 | 5 | 2 | 5 | 6 | 5 | 6 | 2 | 2 | 5 | 6 | 2 |
| | *By 2029 in the US, doctors will transition from the role of dispensers of knowledge to managing teams and information systems.* | | 3 | 2 | 5 | 2 | 7 | 5 | 5 | 5 | 3 | 5 | 6 | 7 | 1 |
| | *By 2029 in the US, AI/ML tools will change the reimbursement structure for routine clinical tasks.* | | 6 | 7 | 4 | 2 | 6 | 2 | 7 | 5 | 6 | 5 | 6 | 6 | 3 |

| | | | | | | | | | | | | | | | |
|---|---|---|---|---|---|---|---|---|---|---|---|---|---|---|---|
| VI. Technological Advancements in Primary Care | *Will improvements in the diagnostic accuracy of AI/ML tools require technological breakthroughs?* | Yes / No | No | No | Yes | No | Yes | Yes | Yes | No | Yes | Yes | No | Yes | No |
| | *If, 'Yes': Improvements in diagnostic accuracy of AI/ML tools will require technological breakthroughs in causal modelling.* | 1 - strongly disagree to 7 - strongly agree | - | - | 6 | - | 7 | 7 | 5 | - | 7 | 6 | - | 7 | - |
| | *If, 'Yes': Improvements in diagnostic accuracy of AI/ML tools will require technological breakthroughs in artificial general intelligence.* | | - | - | 6 | - | 5 | 7 | 5 | - | 7 | 3 | - | 4 | - |
| | *If, 'Yes': Improvements in diagnostic accuracy of AI/ML tools will require technological breakthroughs in the interpretability of certain approaches such as deep learning.* | | - | - | 6 | - | 6 | 7 | 7 | - | 5 | 6 | - | 2 | - |
| | *If, 'Yes': Improvements in diagnostic accuracy of AI/ML tools will require technological breakthroughs in human-level natural language processing.* | | - | - | 7 | - | 7 | 7 | 7 | - | 7 | 5 | - | 7 | - |
| | *If, 'Yes': Improvements in diagnostic accuracy of AI/ML tools will require technological breakthroughs in semi-supervised learning.* | | - | - | 6 | - | 6 | 7 | 7 | - | 7 | 5 | - | 7 | - |
| | *If, 'Yes': Improvements in diagnostic accuracy of AI/ML tools will require technological breakthroughs to harness the sensor data from smartphones and wearables to forecast individual symptom trajectories.* | | - | - | 6 | - | 6 | 7 | 7 | - | 7 | 7 | - | 2 | - |

| | | | | | | | | | | | | | | | |
|---|---|---|---|---|---|---|---|---|---|---|---|---|---|---|---|
| | *To improve the diagnostic accuracy of AI/ML tools, will require improved data quality.* | | 6 | 7 | 7 | 6 | 6 | 7 | 7 | 1 | 6 | 7 | 6 | 7 | 7 |
| | *To improve the diagnostic accuracy of AI/ML tools, will require integrated data sets.* | | 7 | 7 | 7 | 6 | 7 | 7 | 7 | 6 | 7 | 6 | 6 | 7 | 7 |
| | *In the US, regulatory issues in improving diagnostic accuracy of AI/ML tools will be more challenging than technical issues.* | | 7 | 6 | 7 | 5 | 6 | 5 | 7 | 5 | 5 | 4 | 6 | 6 | 7 |
| VII. The Long-term Future of the Profession | *In the US, primary care doctors will be one of the last specialties to be replaced by AI/ML in medicine.* | | 6 | 7 | 7 | 5 | 5 | 2 | 6 | 7 | 5 | 6 | 6 | 3 | 6 |
| | *In the US, primary care doctors will always be required as gatekeepers in medicine.* | | 6 | 4 | 6 | 6 | 5 | 3 | 3 | 3 | 5 | 6 | 6 | 1 | 6 |
| | *In the US, primary care doctors will always be required to synthesize information.* | | 7 | 4 | 6 | 6 | 6 | 5 | 3 | 6 | 5 | 5 | 6 | 2 | 5 |
| | *In the US, primary care doctors will always be required to deliver empathic aspects of care.* | | 7 | 7 | 7 | 6 | 7 | 2 | 5 | 7 | 6 | 5 | 6 | 1 | 5 |
| | *In the US, patients will always prefer humans as gatekeepers of their medical care.* | | 6 | 6 | 6 | 6 | 6 | 2 | 4 | 7 | 4 | 4 | 3 | 1 | 3 |
| | *In the US, adoption of AI/ML tools in health care will be slow due to the culture of medicine.* | | 7 | 6 | 1 | 7 | 6 | 4 | 6 | 7 | 4 | 4 | 3 | 7 | 6 |
| | *In the US, there is a 90% chance that primary care doctors will be obsolete 50 years from now.* | | 1 | 3 | 1 | 1 | 2 | 4 | 6 | 1 | 1 | 1 | 2 | 7 | 2 |
| | *In the US, there is a 90% chance that primary care doctors will be obsolete 100 years from now.* | | 1 | 3 | 1 | 1 | 2 | 4 | 6 | 1 | 1 | 2 | 2 | 7 | 3 |
