# Supplementary material for: US primary care in 2029: A Delphi survey on the impact of machine learning
Source: PLoS One. 2020 Oct 8;15(10):e0239947. doi: 10.1371/journal.pone.0239947 (PMC7544100; doi:10.1371/journal.pone.0239947)
Supplement: Attachment — Submitted filename: Response to reviewers.docx [file pone.0239947.s004.docx]

31 August 2020

Dear Plos One,

Thank you for the excellent feedback and for considering a resubmission of our paper. Please find our response to the reviewers and editor of our manuscript, below.

PONE-D-20-12979
US Primary Care in 2029: A Delphi Survey on the Impact of Machine Learning
PLOS ONE

Response to Editor

In your revised cover letter, please address the following prompts:

a) If there are ethical or legal restrictions on sharing a de-identified data set, please explain them in detail (e.g., data contain potentially identifying or sensitive patient information) and who has imposed them (e.g., an ethics committee). Please also provide contact information for a data access committee, ethics committee, or other institutional body to which data requests may be sent.

Response: Round 1 of the Delphi Poll includes demographic data, and qualitative responses. Because of the sample size, even with de-identification of this data, it would be possible to re-identify the participants thereby posing a risk to respondents’ anonymity. In addition, Round 3 of the Delphi Poll comprised 13 bespoke, individual surveys to each participant. Again, this information cannot be shared publicly because of the need to ensure participant anonymity and confidentiality, as per ethical review. However, de-identified raw data is now available as a supplementary files Appendix 3 for Round 2 of the survey.

b) If there are no restrictions, please upload the minimal anonymized data set necessary to replicate your study findings as either Supporting Information files or to a stable, public repository and provide us with the relevant URLs, DOIs, or accession numbers. Please see [http://www.bmj.com/content/340/bmj.c181.long](https://urldefense.proofpoint.com/v2/url?u=http-3A__www.bmj.com_content_340_bmj.c181.long&d=DwQGaQ&c=WknmpdNpvrlj2B5K1aWVqL1SOiF30547pqSuOmtwXTQ&r=wgxI4b4HECcgbtot50EcGOH4ZydL_8dzncIfN88IkMc&m=UulZy9mm0sMtOR-SqCFl0d6phn6OYHBQ0odEqU-kdCw&s=NF3QYEEUMakf7hn4CG3ZyV-vrSlTEla3HAUnzo5Om64&e=) for guidelines on how to de-identify and prepare clinical data for publication. For a list of acceptable repositories, please see [http://journals.plos.org/plosone/s/data-availability#loc-recommended-repositories](https://urldefense.proofpoint.com/v2/url?u=http-3A__journals.plos.org_plosone_s_data-2Davailability-23loc-2Drecommended-2Drepositories&d=DwQGaQ&c=WknmpdNpvrlj2B5K1aWVqL1SOiF30547pqSuOmtwXTQ&r=wgxI4b4HECcgbtot50EcGOH4ZydL_8dzncIfN88IkMc&m=UulZy9mm0sMtOR-SqCFl0d6phn6OYHBQ0odEqU-kdCw&s=GKh-bwSx4uRlkRSqlp9NEzxBicB5fvGFlPBovCl6xtw&e=).

Response: Please see above.

We will update your Data Availability statement on your behalf to reflect the information you provide.

3. Please include captions for your Supporting Information files at the end of your manuscript, and update any in-text citations to match accordingly. Please see our Supporting Information guidelines for more information: [http://journals.plos.org/plosone/s/supporting-information](about:blank).

Response: now added.

4. Please include a caption for figure 3. (Figure 2 caption included 2x)

 Response: We were unsure about what was being requested. Caption for Fig 3 is now included and highlighted in the text. Please let us know if any further amendments are required. **Fig 3. Predicted changes in diagnostic accuracy in US primary care in 2029**

Additional Editor Comments (if provided):

Dear Authors,
I appreciate a lot your manuscript
Follow reviewer indications you can improve your article

Response: Thank you.

[Note: HTML markup is below. Please do not edit.]

Reviewers' comments:

Reviewer's Responses to Questions

**Comments to the Author**

1. Is the manuscript technically sound, and do the data support the conclusions?

The manuscript must describe a technically sound piece of scientific research with data that supports the conclusions. Experiments must have been conducted rigorously, with appropriate controls, replication, and sample sizes. The conclusions must be drawn appropriately based on the data presented.

Reviewer #1: Yes

Reviewer #2: Yes

2. Has the statistical analysis been performed appropriately and rigorously?

Reviewer #1: Yes

Reviewer #2: Yes

3. Have the authors made all data underlying the findings in their manuscript fully available?

The [PLOS Data policy](https://urldefense.proofpoint.com/v2/url?u=http-3A__www.plosone.org_static_policies.action-23sharing&d=DwMGaQ&c=WknmpdNpvrlj2B5K1aWVqL1SOiF30547pqSuOmtwXTQ&r=wgxI4b4HECcgbtot50EcGOH4ZydL_8dzncIfN88IkMc&m=UulZy9mm0sMtOR-SqCFl0d6phn6OYHBQ0odEqU-kdCw&s=R0wRw061BUqf-EsR8LpbwF-up0ul3HvQUpFL_JZ9TdA&e=) requires authors to make all data underlying the findings described in their manuscript fully available without restriction, with rare exception (please refer to the Data Availability Statement in the manuscript PDF file). The data should be provided as part of the manuscript or its supporting information, or deposited to a public repository. For example, in addition to summary statistics, the data points behind means, medians and variance measures should be available. If there are restrictions on publicly sharing data—e.g. participant privacy or use of data from a third party—those must be specified.

Reviewer #1: Yes

Reviewer #2: Yes

4. Is the manuscript presented in an intelligible fashion and written in standard English?

PLOS ONE does not copyedit accepted manuscripts, so the language in submitted articles must be clear, correct, and unambiguous. Any typographical or grammatical errors should be corrected at revision, so please note any specific errors here.

Reviewer #1: Yes

Reviewer #2: Yes

5. Review Comments to the Author

Please use the space provided to explain your answers to the questions above. You may also include additional comments for the author, including concerns about dual publication, research ethics, or publication ethics. (Please upload your review as an attachment if it exceeds 20,000 characters)

Reviewer #1: Congratulations for the appropriate methodology and for the clarity of your explanation.
Probably the only integration that can be useful in the discussion is the specification of why the shift towards computing and engineering in the educational background of students entering medical school is considered necessary:
- are there any risks if these techniques, which will become standards of care soon, are used in the absence of specific training? If there is any relevant literature please specify, if not it will be an interesting perspective to be pursued by further study.

Response: We thank Reviewer 1 for these kind comments. Great point. While there is no relevant literature on the matter, we have now added the following nuanced point on page 22:

“However, the survey did not reveal whether experts perceived there to be risks of physicians using ML/AL tools without a computing and engineering background, or indeed, without an ethics or evidence-based perspective, on these techniques. The panel’s predictions on education trends should also be observed against the currently limited debate about the need for curricular changes in medical education.(37–39)”.

In addition, to draw out the ethical, as opposed to technical considerations, in the Conclusion we have added 2 additional citations on ethics and AI, to reflect the importance of ethical education to digital literacy among clinicians and the public:

Vayena E, Blasimme A, Cohen IG. Machine learning in medicine: Addressing ethical challenges. PLoS medicine. 2018;15(11):e1002689.

Zuboff S. The Age of Surveillance Capitalism: The Fight for a Human Future at the New Frontier of Power. Profile Books; 2019.

Reviewer #2: Blease et al used a modified Delphi poll to solicit health informaticians’ predictions about the impact of AI/ML on primary care in US in 2029. The text is well-written and the flow is logical. The experiments are well-planned and executed. While there are many interesting questions that arise from this work, e.g. would there be contrasting predictions between medical experts with and without computer science background, the manuscript in its current form tells a coherent story and build a foundation for future work. One minor comment - I think the authors should cite Liyanago et al. ([https://www.ncbi.nlm.nih.gov/pmc/articles/PMC6697547/](https://urldefense.proofpoint.com/v2/url?u=https-3A__www.ncbi.nlm.nih.gov_pmc_articles_PMC6697547_&d=DwQGaQ&c=WknmpdNpvrlj2B5K1aWVqL1SOiF30547pqSuOmtwXTQ&r=wgxI4b4HECcgbtot50EcGOH4ZydL_8dzncIfN88IkMc&m=UulZy9mm0sMtOR-SqCFl0d6phn6OYHBQ0odEqU-kdCw&s=mBPI0BpYcp7Vcceu7E9K_IyvzSPxZjCYfCAwjBeQlnk&e=))

Response: We thank Reviewer 2 for this encouraging feedback. We have now included the citation in the paper [citation 17]. On page 4 we have amended the text as follows:

“Currently, there is scarce exploration of consensus views among informaticians (17); in particular, on how AI/ML might meaningfully influence medical care in the short-term (18).”

6. PLOS authors have the option to publish the peer review history of their article ([what does this mean?](https://urldefense.proofpoint.com/v2/url?u=https-3A__journals.plos.org_plosone_s_editorial-2Dand-2Dpeer-2Dreview-2Dprocess-23loc-2Dpeer-2Dreview-2Dhistory&d=DwMGaQ&c=WknmpdNpvrlj2B5K1aWVqL1SOiF30547pqSuOmtwXTQ&r=wgxI4b4HECcgbtot50EcGOH4ZydL_8dzncIfN88IkMc&m=UulZy9mm0sMtOR-SqCFl0d6phn6OYHBQ0odEqU-kdCw&s=7yD6bGVyge6wCsf8Aw2-PJkCDenxObVLjeZLDr83qWk&e=)). If published, this will include your full peer review and any attached files.


If you choose “no”, your identity will remain anonymous but your review may still be made public.


**Do you want your identity to be public for this peer review?** For information about this choice, including consent withdrawal, please see our [Privacy Policy](https://urldefense.proofpoint.com/v2/url?u=https-3A__www.plos.org_privacy-2Dpolicy&d=DwMGaQ&c=WknmpdNpvrlj2B5K1aWVqL1SOiF30547pqSuOmtwXTQ&r=wgxI4b4HECcgbtot50EcGOH4ZydL_8dzncIfN88IkMc&m=UulZy9mm0sMtOR-SqCFl0d6phn6OYHBQ0odEqU-kdCw&s=tPRMXV2efBk6pUC81TWSsRyIzUqtfu4myrAiKYCUcwI&e=).

Reviewer #1: **Yes:** Calogero Casà

Reviewer #2: No
